# Study on non-bioparticles and *Staphylococcus aureus* by dielectrophoresis†

**DOI:** 10.1039/c9ra05886a

**Published:** 2020-01-15

**Authors:** Qiaoying Chen, Zhongqing Cao, Yong J. Yuan

**Affiliations:** Laboratory of Biosensing and MicroMechatronics, School of Materials Science and Engineering, Southwest Jiaotong University Chengdu Sichuan China yongyuan@swjtu.edu.cn; School of Mechanical Engineering, Southwest Jiaotong University Chengdu Sichuan China zqcao@swjtu.edu.cn

## Abstract

This article demonstrated a chip device with alternating current (AC) dielectrophoresis (DEP) for separation of non-biological micro-particle and bacteria mixtures. The DEP separation was achieved by a pair of metal electrodes with the shape of radal-interdigital to generate a localized non-uniform AC electric field. The electric field and DEP force were firstly investigated by finite element methods (FEM). The mixed microparticles such as different scaled polystyrene (PS) beads, PS beads with inorganic micro-particles (*e.g.*, ZnO and silica beads) and non-bioparticles with bacterial *Staphylococcus aureus* (*S. aureus*) were successfully separated at DEP-on-a-chip by an AC electric field of 20 kHz, 10 kHz and 1 MHz, respectively. The results indicated that DEP trapping can be considered as a potential candidate method for investigating the separation of biological mixtures, and may well prove to have a great impact on *in situ* monitoring of environmental and/or biological samples by DEP-on-a-chip.

## Abstract 2

This article demonstrated a chip device with alternating current (AC) dielectrophoresis (DEP) for separation of non-biological micro-particle and bacteria mixtures.

## Graphical abstract

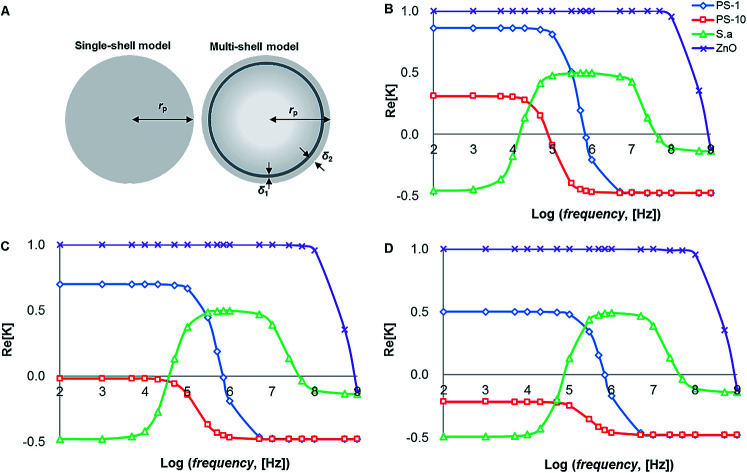

## 1. Introduction

Dielectrophoresis (DEP) forces^1^ are flexible and can be modified simply by changing frequencies, which showed some advantages over other methods, *i.e.*, label-free, ease of handling, high precision and being high speed. DEP has attracted a great deal of attention to control particles due to its high selectivity and rapidity in manipulation.^2^ It is based upon the fact that particles with different electrical characteristics will behave diversely in a non-uniform electric field. It has been used not only for trapping processes,^3^ but also for aligning,^4–6^ isolating and separation various sized particles,^7,8^ and applied in a wide range of medical research,^9,10^ biological^11–14^ and environmental^15^ applications to analyze particles of interest. The particles should be trapped in a detection region before analysis, as the position of the particle has a big impact on the accuracy of the measurement results. DEP-on-a-chip^16^ will be key to developing a rapid, accurate, portable, simple-use and cost-effective microfluidic device with a promising impact in multi-particle manipulating, microfluidics, immune analysis, and micro-total analysis systems (μTAS).

PS beads with stable physicochemical properties are used as numerical analysis objects in this area,^17^ especially to normalize the trapping, enrichment and separation by DEP. Freedman *et al.*^18^ extended tracked 1 μm PS beads at the conditions to DNA trapping, and demonstrated the distinct forces observed for both DEP and electrothermal flow (ETF). Saucedo-Espinosa and Lapizco-Encinas^19^ designed an iDEP device, and illustrated the effects of particle size and shape on DEP trapping by employing 1 and 2 μm PS beads and *E. coli* cells. The results showed that PS beads size and flow speed have a significant effect on the magnitude, location, and shape of DEP trapping regions. In 2017, Allen *et al.*^20^ demonstrated both positive DEP (pDEP) and negative DEP (nDEP) using silver-coated hollow glass spheres and PS beads with the isomotive DEP (isoDEP) devices, respectively. Both alternating current (AC) and direct current (DC) electric fields can trap PS beads by nDEP at approximately 95–100% of trapping efficiency. Moreover, PS beads are often used in a biochemistry field because its sizes are tunably close to cells such as bacteria (0.8–2 μm),^21^ red blood cells (7–8 μm),^22^ liver cells (20–30 μm) and many cancer cells (10–30 μm),^23^ which can provide significant references for manipulation of biological particles. The separation of two groups with a similar size was hard with a fixed frequency.^24^ Su and Voldman^8^ then developed an automated system and presented characterization of the method with 6 and 10 μm PS beads and HL-60 cells, as well as its application to rapidly discriminating neutrophils with different activation states. They used 6 and 10 μm carboxyl-modified PS beads for model validation and calibration. The system accumulated large DEP separation datasets of different cells and label-free applications.

A wide range of frequencies has been investigated from 1 kHz to 500 MHz. Interdigital electrodes and circular electrode arrays were used in literature. The key challenges in DEP are to separate multi-particles at a time. The radial-interdigital electrodes proposed has great potential of separating multi-particles by DEP-on-a-chip. Because of the gradient variation in an electric field, different particles can be in different regions by DEP forces. The intention of this work was to *in situ* examine the interplay between PS beads with inorganic micro-particles and bio-particles, by determining DEP trapping percentages over a broad spectrum of frequencies. Experiments were carried out in the orientation of particles in frequency-selectable directions.^25^ In the whole process, the vibration of the particle chain is very small due to Brownian motion.^26^

Here, we presented a device with AC electric fields by a non-uniform microelectrode array, along the direction of electric-field intensity by much lower frequencies and applied voltages to pattern and separate particles. It was demonstrated respectively in separation of colloidal PS beads by size, PS beads and inorganic micro-particle (silica and ZnO beads) and bio-particles (*S. aureus*). It will provide a new method of diagnosis of *S. aureus* in a complex environmental sample without separation, purification and cell-culture processes. It can also improve the purity of *S. aureus*, by avoiding the pollution of particles during operation.

## 2. Theory

### 2.1. DEP force

Unlike DC,^27^ AC electric fields can be used to manipulate many types of particles in different media by simply adjusting AC parameters (*i.e.*, magnitude, frequency, wave shape, wave symmetry and phase, *etc.*), while eliminating the influence of linear electrokinetic phenomenon including the traditional electrophoresis and electroosmosis. DEP induces chaining of similar particles parallel to the local field direction, independent of whether particles exhibit pDEP or nDEP.^4,6^ The DEP force for particles with spherical geometries is given by

1 ***F***_DEP_ = 2π*r*_p_^3^*ε*_m_Re[*K*(*ω*)]∇***E***^2^

2
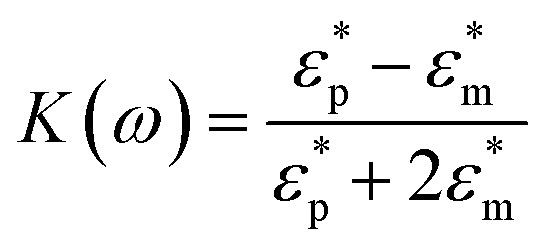


The Clausius–Mossotti (CM) factor *K*(*ω*) is related to the electric field frequency (*ω* = 2π*f*), the conductivity (*σ*) and complex permittivity (ε*) of particles and suspending medium (ε* = *ε* − *jσ*/*ω*, *j*^2^ = −1), subscripts p and m mean particles and suspending medium, respectively.^28^ The *K*(*ω*) can theoretically have a value from −0.5 to 1.0. According to the above equations, the sign of the *K*(*ω*) determines whether a particle is repelled from or attracted to regions of high electric field strength. A positive factor indicates attraction, generally referred to as pDEP.

The basic principle of DEP depending on the extent of polarization is described. In time averaged translational DEP, both *K*(*ω*) and DEP force are frequency-dependent. The crossover frequency (COF) is that frequency at which a particle subjected to DEP transitions from pDEP to nDEP or from nDEP to pDEP.^7^ Thus, the crossover angular frequency, *ω*_c_, is calculated by letting Re(*K*) = 0 for *ε*_p_ < *ε*_m_ and *σ*_p_ < *σ*_m_, which may be defined as:^29^3
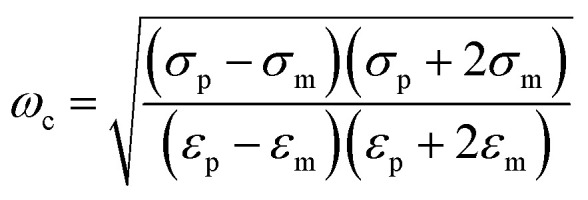


For low permittivity particles in aqueous media (*e.g.*, deionized (DI) water), particles move toward the high field region at *ω* < *ω*_c_, referred to as pDEP and toward the low field region at *ω* > *ω*_c_, referred to as nDEP. For particles in water, nDEP occurs when the permittivities of the system dominate due to the large *ε* = 79 of water compared to nearly all other substances. The COF is given by4
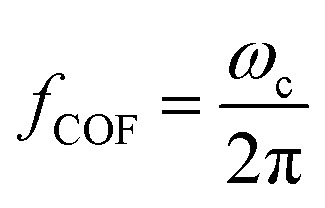


Here, *f*_COF_ is known as the crossover frequency where DEP force is zero (Re(*K*) = 0). The key parameter, electrical conductivity of a solid homogeneous PS bead, can be expressed as5
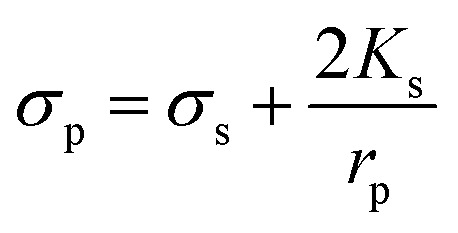
where *K*_s_ represents the surface conductance and *r*_p_ is the radius of the particle.^30^ The non-uniform surface conduction of counterionic charges within the induced double layer was formed right on the particle surface.^31–33^ It can be obtained by enabling an electrical current balance at the outer edge and inner edge of the electric double layer, as long as the surface gradient of the surface conduction current is taken into account, namely the Dukhin number.^32,33^

The value of *K*_s_ has a linear relationship with the PS bead diameter if the permittivities of suspending medium and PS beads are constantly undergoing *f*_COF_. The surface conductance has been calculated for PS beads of different diameters.^34^ In DEP separation, the variables of voltage, frequency and buffer conductivity need to be considered.^35^ This study focused on the characterization of particles in behaviors by frequency.

### 2.2. Particle geometry factor

Because of the different characteristics of PS particles and cells, there are significant differences in morphology and dielectric properties, as shown in Fig. 5A. PS particles are homogeneous particles while cells are heterogeneous particles. The conventional inorganic particles can be equivalent to the single shell model with radius *r*_p_. Cells are different from the conventional inorganic particles, and they need to be regarded as multi-shell models. Thus, *S. aureus* can be regarded as an equivalent sphere with bilayer membrane structure^36^ (radius is *r*_p_): cell plasma membrane thickness *δ*_1_ is 8 nm, cell wall thickness is 20 nm.

### 2.3. Conductivity factor

The frequency is one of the important factors to ensure the polarizability of PS beads, while the voltage determines the stable and sufficient DEP forces being applied to the induced dipole moment, which depend on the voltage and electrode geometry. Since the DEP technique directly affects cell physiology, several electro-physiological effects need to be considered when designing the electrode geometry.

**Fig. 1:**
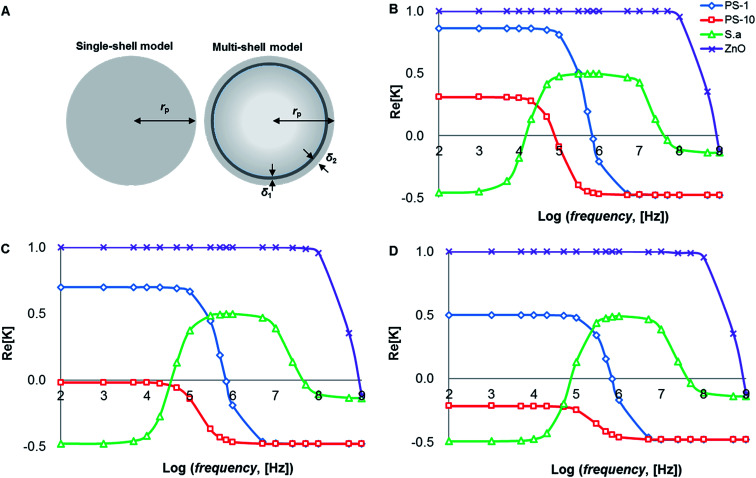
(A) Illustration of homogeneous particle and inhomogeneous cell. Re[*K*] of different particles as a function of frequency in media with conductivities of 0.2 mS m^−1^ (B), 0.5 mS m^−1^ (C) and 1 mS m^−1^ (D), respectively.

Theoretical predictions of suspension media's conductivity dependency (0.2 mS m^−1^, 0.5 mS m^−1^ and 1 mS m^−1^) of Re[*K*(*ω*)] is shown in Fig. 1, frequency ranges were from 1 kHz to 10 MHz while electric potentials applied at 8 V peak-to-peak (*V*_pp_) amplitude. Other parameters were summarized in Table 1.

**Table 1:** Parameters in the model

Particles	Diameter (μm)	Density (g cm^−3^)	Conductivities (mS m^−1^)	Relative permittivity
PS-1 (ref. 37)	1	1.05	4	2.55
PS-10	10	1.05	0.47	2.55
Silica^38^	1–2	2.2	—	3.9
ZnO^39^	1	5.606	5	1.7
*S. aureus* (live)^40,41^	∼1	∼1	0.75 (cytoplasm), 2.5 × 10^−4^ (membrane)	60 (cytoplasm), 6 (membrane)
*S. aureus* (dead)^42,43^	∼1	∼1	0.75 (cytoplasm), 1 (membrane)	60 (cytoplasm), 6 (membrane)
Suspension			0.2 (DI water), 0.5 (sucrose), 1 (sucrose)	78

The spectra are very different for live/dead bacteria, reflecting their morphological and dielectric specific properties. Based on the calculation in Fig. 1B, pDEP (Re[*K*(*ω*)]>0) for 1 μm PS beads was predicted below 1 MHz, while 10 μm PS beads below 100 kHz. The pDEP behavior of ZnO beads occurred approximately below 1 GHz, while *S. aureus* was expected between 10 kHz and 60 MHz. If medium conductivity was 0.5 mS m^−1^ as shown in Fig. 1C, the pDEP behaviors of both 1 μm PS and ZnO beads were almost the same as the situation of 0.2 mS m^−1^ as shown in Fig. 1B. The pDEP behavior of *S. aureus* was slightly narrowed between 60 kHz and 60 MHz. When the medium conductivity was further increased to 1 mS m^−1^ in Fig. 1D, the pDEP behaviors of both 1 μm PS and ZnO beads were very close to that of Fig. 1B and C, except for lowering Re[*K*(*ω*)] magnitudes of 1 μm PS beads.

The pDEP behavior of *S. aureus* was expected to be between 90 kHz and 60 MHz as shown in Fig. 1D, and the nDEP behaviors of PS beads were predicted by energizing an electric field at 1 MHz frequency as determined by negative values of theoretical Re[*K*(*ω*)] calculated. If the frequency was set to above 1 MHz, a strong pDEP force could be obtained for *S. aureus* bacteria trapping, which eliminating PS beads due to their nDEP behaviors. The relative negative polarizability of induced dipole is enhanced due to a decrease in particle surface conductivity, thus increasing the magnitude of nDEP force field. The overall comparison of the calculated results of Re[*K*(*ω*)] provides a separation possibility of biological mixtures.

## 3. Simulation and experiments

### 3.1. Design and simulation

**Fig. 2:**
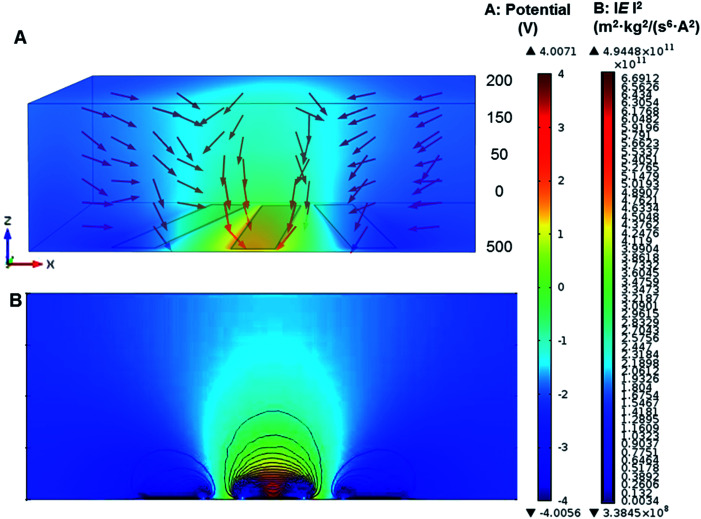
Simulation of electric field and direction of DEP forces for 1 μm PS beads. (A) The electric field distribution and direction of DEP forces for 1 μm PS beads (*f* = 10 kHz) in *z* axis. (B) The contours of the squared electric field distribution (m^2^ kg^2^ (s^6^ A^2^)^−1^) in cross section, and the highest value occurred near electrode's edge.

Microelectrode architecture is one of the important factors to ensure stable and sufficient DEP forces being applied to the induced dipole of the target particles suspended in the liquid medium. The geometry of microelectrodes was designed for specific investigation purpose. A numerical simulation was created to find estimation of the field intensity and the location of electric field maxima, and then verifying the cell trapping locations predicted. Here, a 3D AC/DC electrostatic model of a non-uniform electric field in aqueous solution was performed for analysis of electric field distribution and prediction of trapping location of different particles by finite element multiphysics software COMSOL Multiphysics 4.2a (Burlington, MA, USA). As shown in Fig. 2, microelectrodes were radial-interdigital strips of 60 μm wide and 800 μm long, separated by gaps from 50 to 200 μm. The red vectors in Fig. 2A show the direction of DEP forces (not in scale). The gap between electrode strips generates a powerful nDEP force due to the Laplace equations with fixed potential. The direction of the nDEP force is downward (as shown by red arrows), which drives particles to get stuck at the bottom. The electric field and contour of DEP forces for 1 μm PS beads (*f* = 10 kHz) were shown in Fig. 2B. The gap between electrode strips and the middle of them are dominated by nDEP, while pDEP occurs near the strips' edges.

The electric field distribution in a non-uniform field was initially derived from Laplace's equation. By taking the gradient of the square of electric field intensity, the relative strength of the DEP force was calculated. The electrode gap was designed to generate low electric fields so as to move the particles to the gap by nDEP.^44^ In addition, the strength of the DEP force was also affected by the conductivity and permittivity of particles and media.

### 3.2. Device fabrication and assembly

The DEP chip was microfabricated using a standard photolithography on a 3-inch glass wafer for its transparency and low-cost. The wafers were cleaned by surfactant solution for 30 min and then hot piranha solution (70% sulfuric acid, H_2_SO_4_ and 30% hydrogen peroxide, H_2_O_2_) for 6 h. A 400 nm layer of Al was deposited in an electron-beam evaporator. Al-coated wafers were vapor-primed with hexamethyldisilazane (HMDS) for 30 min, followed by spin-coating of 1.2 μm positive photoresist (AZ 1500, AZ Electronic Materials Co., Ltd., Shizuoka, Japan) with a spin coater (WS-400B-6NPP-LITE, Laurell, Mycro Technologies, Wuhan, China) for 30 s at 3000 rpm. The photoresist coated wafer was then pre-baked for 90 s at 100 °C and cooled for 2 min before UV light exposure by a mask-aligner (DS-URE-2000/25-1, Chengdu Institute of Optic and Electronics, Chengdu, China) followed by post-baking for 60 s at 120 °C. After the post-bake process, the wafer then underwent a developing process where the whole wafer was immersed inside a developer. The uncovered Al film was then etched using wet-etching (H_3_PO_4_ : HAc : HNO_3_ : H_2_O = 19 : 1 : 1 : 2, v/v) for 5 min. The micro-electrodes on the wafer were realized after soaking with acetone in an ultrasonic bath. The wafers were diced (ADT 7100, Advanced Dicing Technology, Inc., Yokneam, Israel) and cleaned with DI water before dried with nitrogen. DEP-on-a-chip assembled in a holder, micro-electrodes were connected externally to a function generator (Agilent 33250A, Agilent Technologies, Inc., Santa Clara, CA, USA) *via* copper tape. The particles were suspended in solution and subjected to AC electric field induced by Al micro-electrodes.

### 3.3. Chemicals and materials

Monodispersed PS beads of various sizes (diameter 1–10 μm) were purchased from Spherotech Inc. (Chicago, IL, USA). DI water (Omni Analytic Inc.) was used in preparation of all aqueous solutions. 1 μm ZnO and silica beads were purchased from Buhan Chemical Technology Inc (Shanghai, China). *S. aureus* was from Jiangsu Provincial for Disease Control and Prevention (Nanjing, China). *S. aureus* cells were cultured on Colombian sheet blood plate in an incubator (Thermo Fisher 3111, Thermo Fisher Scientific Inc., Marietta, OH, USA) and allowed to grow overnight at 35 °C.

The evaluation of suspension medium conductivity *σ*_m_ was conducted by adding sucrose (99%, Sigma-Aldrich, Saint Louis, MO, USA) to PS beads suspensions. The conductivity was varied from 0.2 to 1 mS m^−1^ (added by several microliters 2 mM PBS buffer), measured by a conductivity meter (HI8733, Hanna, Italy). The particle mixture of Sam-A was prepared by suspending 1 and 10 μm PS beads in DI water with a final concentration of ∼10^7^ particles per mL. Sam-B was prepared by suspending 1 μm PS beads, ZnO and silica beads in DI water (∼10^7^ particles per mL). The bio-mixture of Sam-C was prepared by suspending *S. aureus* cells and Sam-B in DI water (∼10^7^ particles per mL).

Live *S. aureus* cells were heated at 80 °C for 30 min to be programmed cell death. Live and dead cells were stained using methylthionine chloride^45^ (Nobleryder, Beijing, China), respectively. In addition, 5 μL dye was mixed with 500 mL bacterial suspension, incubated at room temperature in the dark for 10 min. The mixture was centrifuged for 4 min at 1500 rcf, washed two times, and then suspended in 0.1 mM PBS buffer to achieve target concentrations of approximately 10^7^ cells per mL. The total amount of cells was counted by a blood counting chamber. The final conductivity was 0.2 mS m^−1^.

The manipulation process was monitored and recorded by upright optical microscopy (DM 2500, Leica, Germany) equipped with a CCD camera (7100, Leica, Germany). The motion of particles and cells was recorded at 10 frames per second in horizontal direction, and the average levitation heights for beads were measured by calibration of a microscope focus dial. Labeled bacteria were introduced into the DEP chip by pipetting 10 μL of particles suspension. If the applied voltage was higher than 6 V (meanwhile *f* < 1 kHz), electrochemical dissolution of Al electrodes would occur.

## 4. Results and discussion

All DEP data were obtained under standardized operating conditions. A functional generator was used to trigger a sine wave of 8 V peak-to-peak (*V*_pp_) voltage. The manipulation for a duration of 5 min was repeated from 100 Hz to 70 MHz. The particles were trapped at the electrode edges or gaps at several frequencies per decade, and DEP spectra were thus obtained.

### 4.1. Electric-field distribution and DEP force orientation

#### 4.1.1. Electric field distributed vertically

**Fig. 3:**
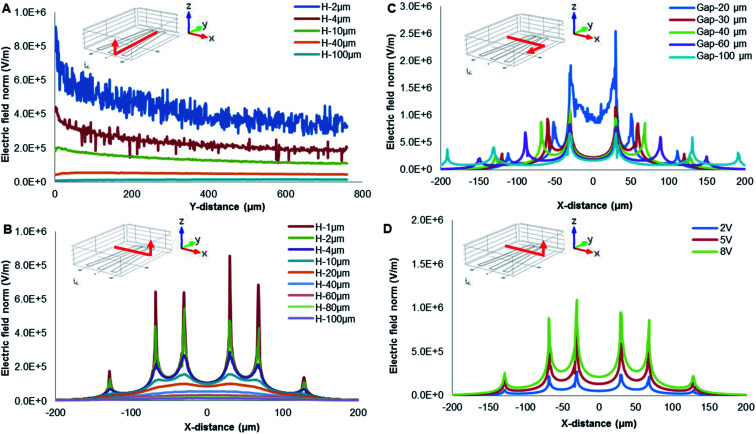
Electric field configurations of DEP simulation. (A) Electric-field distribution on the *Z*-axis in the *Y*-direction; (B) electric-field distribution on the *Z*-axis with gaps in the *X*-direction; (C) electric-field distribution on the *Y*-axis in the *X*-direction; (D) electric-field distribution on the *Z*-axis different voltages applied at 200 μm gap in the *X*-direction.

The effect of heights in the *Y*-direction on electric distribution of different field locations was presented as shown in Fig. 3A. The electric field intensity decreased along the gap width with increasing between electrodes, the highest value generated when the gap width is the least (*i.e.*, closed to the center of radial-interdigital electrodes). The electric field intensity was almost negligible above 40 μm high. The distribution of electric fields in the *X*-direction was demonstrated in Fig. 3B. As clearly indicated, the high electric-field strength was close to the electrodes' surface, especially at their edges. The effect of gap's spacing on electric-field distribution would be further investigated to evaluate DEP-on-a-chip.

#### 4.1.2. Electric field vertical distribution by different horizontal gaps

The effect of electrode's gaps in the *X*-direction on surface electric distribution was presented in Fig. 3C. The different cross-sections were set at 20, 30, 40, 60 and 100 μm gaps (as *Y* = 0, 80, 160, 360, 760 μm) in the *X*-direction. Apparently, the electric field intensity is inversely proportional to the width of gaps. If gap's width was 20 μm, the electric field intensity was the strongest, and its value was more than twice that of the 30 μm gap contributed.

#### 4.1.3. Electric-field domination

As demonstrated in Fig. 3A–C, architecture of DEP-on-a-chip played a significant role of electric-field distribution. According to eqn (1), DEP force is proportional to ***E***^2^, the potential applied by an external electric field. The voltages applied would therefore affect the electric field strength of the DEP force field. As shown in Fig. 3D, electric fields were illustrated at a 200 μm gap in the *X*-direction with different voltages (2 V, 5 V and 8 V). The magnitude of electric fields is obviously increased with increasing voltages applied.

In addition, DEP focusing behavior is mainly dominated by a high magnitude of voltages. The particles on a DEP chip could be manipulated to repel away from electrodes by varying the potential.

#### 4.1.4. Frequency contribution

Apart from DEP-on-a-chip potential discussed above, the particle size and frequency-dependent Re[*K*(*ω*)] as presented in eqn (1) have significant effects on the DEP force, which is proportional to field intensity gradients. As is evident from eqn (2), *K*(*ω*) is related to the electric field frequency in particular, the conductivity and complex permittivity of particles and suspending media. The variation of polarizability of target particles, depending on the frequency of the applied electric field and the particle size, has been extensively discussed in literature on DEP force profiles.

**Fig. 4:**
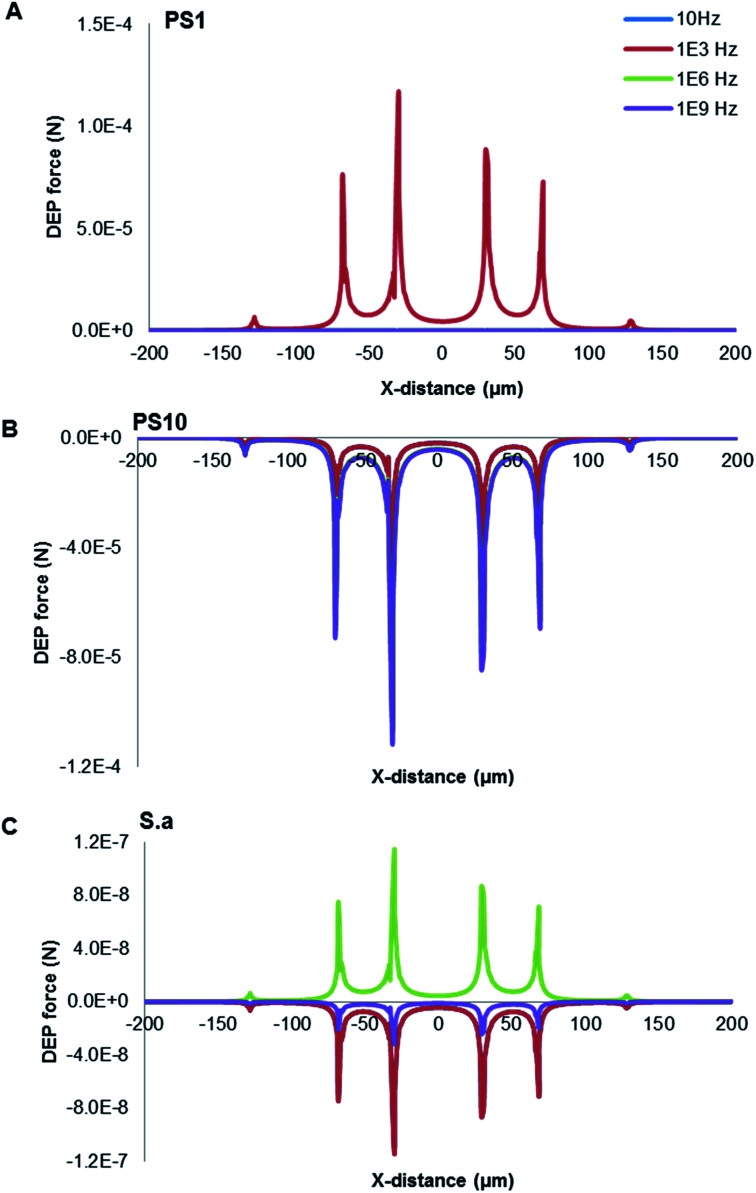
DEP force distribution over the electrode plane at different frequency in the *X*-direction distance. (A) 1 μm PS beads; (B) 10 μm PS beads; (C) *S. aureus*.

As presented in Fig. 4, calculations of DEP force for three typical cases (A) 1 μm PS beads, (B) 10 μm PS beads and (C) *S. aureus* were frequency dependent as predicted at 8 V. The conductivity and permittivity values of the different cell compartments deduced are in broad agreement with those found in literature.^36^ The relative parameters used of target particles and media were summarized in Table 1. The sharp peaks in the DEP force occurred at the edges of electrodes, coinciding with the location of the high field intensity gradients. The 1 μm PS beads experienced the strongest pDEP force at the frequency of ∼1 kHz, with 10 μm PS beads behaving nDEP force. Interestingly, *S. aureus* was provided with both pDEP and nDEP forces. It is exhibited the strongest pDEP force at ∼1 MHz, with the strongest nDEP force at ∼1 kHz. The frequency spectra are very different due to distinguishable characteristics of both PS beads and *S. aureus*, especially reflecting their morphological and dielectric specific properties. The Maxwell–Wagner theory is applicable for a diluted system of homogenous spherical particles, which is very sensitive to the cell membrane permittivity and conductivity. PS bead is a homogeneous particle, while *S. aureus* is a heterogeneous particle. The multi-shell model has been often considered for theoretical estimation of the CM factor for micro-organisms such as *S. aureus*. Charges could therefore be induced by an external electric field along a conductivity and/or permittivity gradient (either structural interfaces or inhomogeneous material). Since both induced charge and applied electric-field oscillate at the same frequency of the voltage signal, their product has a non-zero time-averaged value, resulting in a net DEP force being proportional to the field strength squared even in an AC field.

### 4.2. The characterization of particle motion

**Fig. 5:**
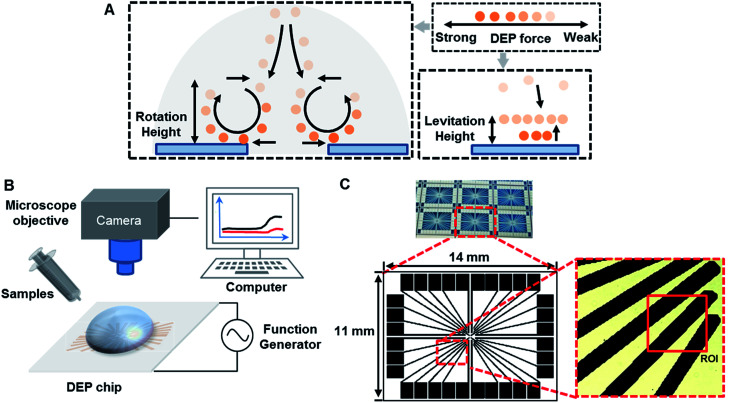
(A) Schematic diagrams of the mechanism of particles trajectories in the lateral direction. (B) Schematic diagram of a DEP-setup system. (C) Images of micro-electrodes fabricated and regions of interest (ROI) on a DEP chip.

The DEP direction of particle motion depends on polarization properties of both particles and suspend medium. Trajectories of particle motion are very important to conduct particle separation. The characterization of particle motion can be further classified into two categories, such as rotation and levitation, as shown in Fig. 5A.

#### 4.2.1. Particle rotation

The rotation is based on pDEP and physical mechanism AC electroosmosis (ACEO),^46^ resulting in recirculating motion (∼1 s per cycle) around an axis oriented parallel to two micro-electrode edges (as shown by arrows in Fig. 5A). The direction of pDEP force is downward near the edge, which drags the particles to the edges of micro-electrodes. The convection of particles from the edges of micro-electrodes – where the field gradient is the highest – to their center is expected to result in a drop in the trapping percentage, as a hydrodynamic force can more easily dominate the weakened DEP. The particles move down from the bulk into the inter-electrode gap and out along the surfaces of the micro-electrodes, with a velocity that varies with frequency. The rotation height results from the resultant forces acted on particles in both vertical and transverse direction, which include gravity, ***F***_ACEO_ and ***F***_DEP_. For the particles with the same size, the differences of gravity are affected by density, while ***F***_DEP_ is affected by permittivity and conductivity of particles in the same suspension.^47^

Initially, the appearance of the low frequency dispersion is surprising since the DEP force saturates in this frequency range to a constant value. The reduction in particle trapping occurs for the onset of ACEO by further examination, which is the origin of the dispersion. In this case, the particles showed the induced vortices by ACEO above the electrode edges, due to move from regions of high electric field (*i.e.*, at the edges of the electrodes) to regions of low electric field (*i.e.*, in the middle of the electrodes). The reduction in attractive pDEP force then makes it easier to shear the particles away from the electrode array, resulting in a decreased trapping amount.^48^

#### 4.2.2. Particle levitation

The levitation is based on nDEP and gravity, which elevates the particles moved away from the electrodes as shown by the arrow in Fig. 5D. Additionally, ACEO is not apparent since this frequency is generally above 20 kHz. When the frequency increased, particles near the gap that undergo nDEP will migrate to the center of the electrode, and then be levitated several micrometers above the surface of the electrode chip.^49^ The levitation height results from the resultant acting force on particles in a vertical direction, such as nDEP and gravity. Efficient positioning of outlets for different particles depends on the accurate prediction of particles' levitation height. For example, particles were separated from a mixture by levitating them at different heights by nDEP^50^ in some microdevices.

#### 4.2.3. Manipulation of particle motion

**Fig. 6:**
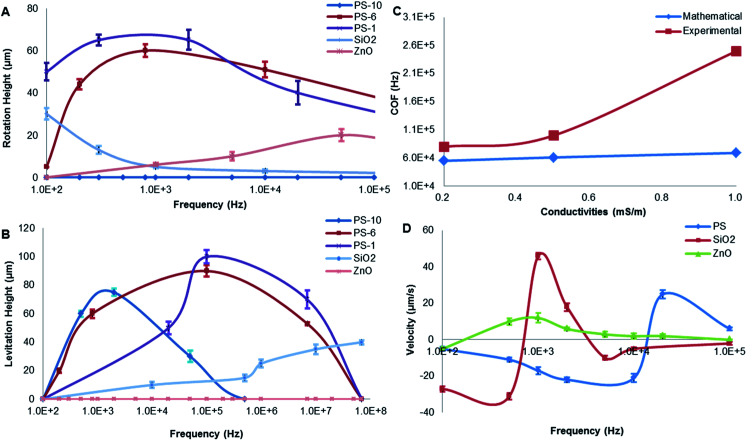
Particle-motion behaviours (*n* = 4). (A) The rotation height of PS beads (10 μm, 6 μm, 1 μm), silica (1 μm) and ZnO beads (1 μm); (B) the levitation height of PS beads (10 μm, 6 μm, 1 μm), silica (1 μm) and ZnO beads (1 μm); (C) comparison of mathematical and experimental results at different conductivities of 0.2 mS m^−1^, 0.5 mS m^−1^, 1 mS m^−1^; (D) the velocity of different particles in ROI (8*V*_pp_, 0.5 mS m^−1^).

The relative contributions of ACEO and DEP forcing on particle trapping would be characterized as a function of frequency. The relevant forces should be considered by choosing the optimal separation frequency and medium. DEP-on-a-chip with micro-electrodes was placed on a microscope platform (Fig. 5B). A close-up of an interesting region was shown in Fig. 5C for investigation of different particles. A sinusoidal wave of 8*V*_pp_ was applied to DEP-on-a-chip micro-electrodes array with the micro-electrode gaps from 40 to 80 μm. The characterizations of different particles in terms of both media conductivity and AC electric field frequency (ranging from 100 Hz to 100 MHz) were presented in Fig. 6.

#### 4.2.4. Significances of pDEP and nDEP

Particle motions were recorded by a calibrated camera. The rotation height of particles can be estimated microscope focusing adjustment by focusing adjustment.^51^ The particle trapping capability of the proposed approach was to demonstrate by a drop (20 μL) of diluted particles (1 μm in diameter) suspension with 0.5 mS m^−1^ conductivity, as shown in Fig. 6A. In addition, 1 μm PS beads undergoing pDEP when the frequency below 1 MHz, the rotation height was approximately 65 μm at 1 kHz. If the size of PS beads increased, the rotation height decreased with increasing frequency. Furthermore, 10 μm PS beads obviously undergoing nDEP, its rotation was disappeared, and the beads were moved away from micro-electrodes. The rotation height of silica beads also decreased with increasing frequency. By contrast, the rotation height of ZnO particles increased with increasing frequency. The resultant forces acted on micro-particles were summarized in Table 2.

**Table 2:** The resultant forces acted on particles

*f*	PS-1	PS-10	Silica	ZnO	*S. aureus*
1 kHz	pDEP + ACEO	nDEP	nDEP	nDEP + ACEO + gravity	nDEP
10 kHz	pDEP + ACEO	nDEP	nDEP	pDEP + ACEO + gravity	pDEP + ACEO
100 kHz	nDEP	nDEP	nDEP	pDEP + gravity	pDEP
1 MHz	nDEP	nDEP	nDEP	pDEP + gravity	pDEP
10 MHz	nDEP	nDEP	nDEP	pDEP + gravity	pDEP
100 MHz	nDEP	nDEP	nDEP	pDEP + gravity	nDEP

The rotation height of PS beads is inversely proportional to particle size under 5 kHz. Compared with 1 μm PS beads, the lower rotation height of 10 μm PS beads may be due to the low DEP force produced by weak polarization. It is likely that the bulk conductivity of the particle plays a more significant role in the total conductivity than that of just the surface as suggested in the literature. In addition, buoyancy and gravity were canceled out.^38,52^ A similar relationship exists between PS beads and silica: the rotation height of silica is smaller than that of PS beads because of the gravity acted on opposite direction. The rotation height of PS beads and ZnO particles in opposite directions were mainly due to the direction of DEP, as shown in Table 3.

**Table 3:** Comparison of DEP forces by different heights above electrodes and particle gravities

*f* = 1 kHz (pN)	PS-1	PS-10	Silica	ZnO	*S. aureus*
H-2μm	365.84	−250 056.68	—	522.23	−250.89
H-10 μm	27.21	−18 596.53	—	38.84	−18.66
H-40 μm	6.68	−4563.45	—	9.53	−4.58
H-100 μm	0.52	−291.61	—	0.61	−0.29
Gravity (pN)	4.40	4400	23.5	73.7	4.61

The levitation height of particles as a function of frequency was shown in Fig. 6B. In addition, 10 μm PS beads were elevated up to 80 μm at 1 kHz and then moved away from the electrodes. Furthermore, 5.6 μm PS beads were levitated to 88 μm if the frequency was increased up to 100 kHz. Then, 1 μm PS beads were further levitated up to 100 μm (a highest point) at 100 kHz. When the size of PS beads was decreased, levitation was enhanced due to nDEP. With increasing particle size, the relative negative polarizability of induced dipole is enhanced due to a decrease in particle surface conductivity, thus increasing the magnitude of nDEP force field. A larger vertical distance from the electrode surface is therefore required for achieving a force balance, due to the gravity being affected by size. The utilization of a force balance between nDEP force and downward gravity can derive an analytical solution for the field squared divergence needed for realizing a steady particle stagnation.

By contrast, the levitation height of silica beads was increased with increasing frequency. ZnO beads were not levitated, and they are mainly due to gravity and the direction of DEP force. When the frequency ranged from 100 Hz to 1 GHz, ZnO beads experienced pDEP, as indicated in Table 2. The pDEP feature of ZnO beads would result in a non-levitation effect. The DEP force acting on gravity therefore levitates different particles to respective heights, potentially providing a vertical-separation. Depending on their vertical locations, the particles ultimately would result in practical horizontal-separation by DEP-on-a-chip architectures.

#### 4.2.5. Effect of crossover frequency

The mathematical and experimental evaluations of COF with three different buffer conductivities were conducted at 8*V*_pp_, as shown in Fig. 6C. The mathematical COF of PS beads was below 300 kHz, indicating that PS beads exhibited nDEP above 300 kHz. The experimental value of COF obtained was a little higher than the calculated value within a low conductivity range. The experimental value increased with increasing conductivity was even larger than that of mathematical. The difference between mathematical and experimental could be due to deviation between theory and experiment, especially at high conductivities. When the conductivity increases, the buffer around particles becomes more polarized, which leads to the double electric layer. The decrease of conductivity difference between PS beads and buffer resulted in the reduction of polarization. The COF of PS beads occurs only when the frequency increases significantly. The experimental values were therefore higher than those calculated. Moreover, electric polarization influence and the possible presence of the dispersion effect which appears at frequencies below a few kHz are not included in the present model.

Distinct COF plays a key role in separating particles. Increasing suspending conductivity (*σ*_m_) can increase COF value, even reverse its sign at low frequencies from positive to negative. However, Al substrate of DEP micro-electrodes experienced electrochemical corrosion at high conductivity solution. The suspending medium with conductivity of 0.5 mS m^−1^ was chosen for further experimental study. In DI water, COFs of 10 μm PS, 1 μm PS 1 μm silica and ZnO beads were, respectively, ∼3 kHz, 20–80 kHz, 5–10 kHz and 50–70 kHz. The COF of live cells was 70 kHz and 40 MHz, it experienced pDEP in the frequency range of 70 kHz to 40 MHz. The COF of dead cells was ∼60 kHz and 10 MHz, experiencing pDEP in the frequency range of ∼60 kHz to 10 MHz.

#### 4.2.6. Particle flow velocity

Digital images captured of particles motion were utilized to calculate the DEP velocity of different particles. Three profiles of velocity *vs.* frequency were PS, silica and ZnO beads, respectively, based on the same electric field and similar particle size (1 μm) as shown in Fig. 6D. Within 100 Hz to 100 kHz frequency range, particle velocities all experienced (1) switching from negative to positive and (2) reaching a maximum at a specific frequency. In particular, particle velocities of both PS and silica beads increased with increasing frequency, either negatively or positively.

The PS beads' assembly rate increased as the frequency increased from 100 Hz to 8 kHz, then increased from negative to positive as the frequency increased from 8 kHz to 20 kHz referring to pDEP, and finally decreased as the frequency higher than 20 kHz by nDEP. By contrast, velocity of silica particles was increased when the frequency up to 1 kHz by pDEP, and then decreased dramatically with increasing frequency. The overall comparison of both PS beads and silica particles of their velocity and frequency indicates a similar trend.^53^ However, ZnO beads presented stable velocity by pDEP. As discussed in the significances of pDEP and nDEP, particles' horizontal-separation by DEP-on-a-chip architectures could be achieved due to practical manipulation of particles' flow velocity. The magnitude of DEP force (pN) was very large near the surface (*H* = 0–2 μm), and the rotation occurs dominantly by pDEP and ACEO if the frequency is below 10 kHz.

### 4.3. The separation of particles

All particles would sediment at the bottom surface of a micro-electrodes array or dissipate in a suspending medium, respectively, due to gravity or the Brownian motion without applying an electric field. Different particles in size are supposed to have individual DEP force as indicated in eqn (1) as a cubed radius term. Based on the polarization properties, particles can be separated by either positive, negative or using both DEP.

#### 4.3.1. Separating multiple-sized organic PS beads

**Fig. 7:**
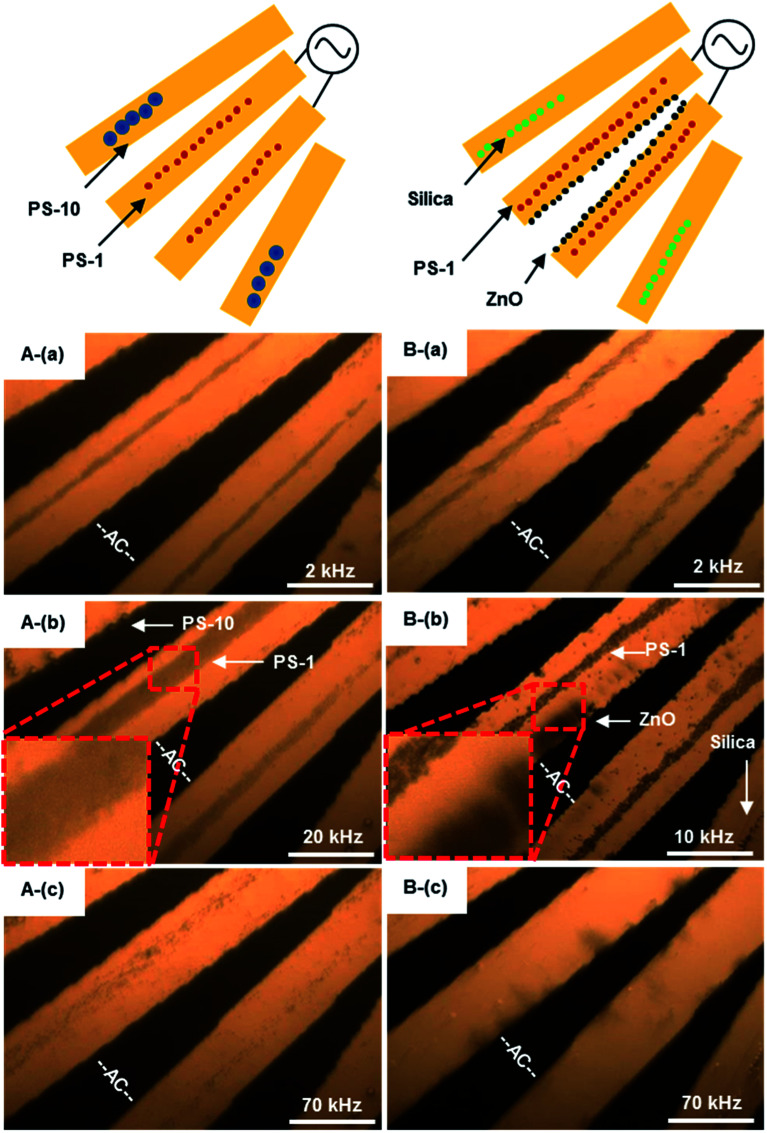
Images of DEP phenomena. The AC signal frequency range 10 kHz to 40 MHz with amplitude of 8*V*_pp_. (A) The frequency-sequenced separation of 1 and 10 μm PS beads, (a) 2 kHz, 1 μm PS beads moving to the electrode center, (b) 20 kHz, 1 μm PS beads concentrated at the electrode center, (c) 70 kHz, PS beads moved away from electrodes. Multimedia view: Fig. 7A.avi;† (B) the frequency-sequenced separation of 1 μm PS beads, ZnO and silica beads, (a) 2 kHz, PS and ZnO beads moved to the electrode center, while silica beads moved away from electrodes, (b) 10 kHz, PS beads concentrated at the electrode center, ZnO beads rotating around the electrode edge, (c) 70 kHz, all non-bioparticles moved away from electrodes. The white scale bar indicates 50 μm. Multimedia view: Fig. 7B.avi.†

A drop (Sam-A) of 20 μL diluted PS beads (1 and 10 μm mixing) suspension was added on the surface of a DEP chip. DEP images were captured by a Leica optical microscopy with a 20× objective and a spot size of 500 μm diameter. As demonstrated in Fig. 7A(a), PS mixing beads moved differently at a frequency range from 2 to 70 kHz. Theoretically, 1 μm PS beads were expected at pDEP below 100 kHz. The distribution of two-sized beads was random. At *f* = 2 kHz, most of the PS beads were accumulated at the center of two connected micro-electrodes, while 10 μm beads rotated around the edges of the micro-electrodes, as shown in Fig. 7A(a). The result was similar to that by Chen,^54^ the levitation height of a specific PS bead strongly depends on the combined contribution of parameters (*i.e.*, frequency, dielectric properties of PS bead and suspension medium). As the frequency increased to 10 kHz, 1 μm PS beads further concentrated at the center of micro-electrodes, as shown in Fig. 7A(b). In addition, 10 μm PS beads started to be dragged to the outer region of the electrodes, as the levitation height is dependent on the *K*(*ω*). The 10 μm PS beads had a lower levitation height than their 1 μm counterparts since they were farther from the micro-electrode tips, due to experiencing a weaker DEP force in *z* direction. Increasing frequency to 70 kHz PS beads moved away from micro-electrodes by nDEP, as shown in Fig. 7A(c) (see Fig. 7A.avi in ESI†). The amount of PS beads accumulated within the captured zone mainly depended upon both magnitude and duration of applied potential (*V*_pp_). The frequency at 20 kHz was the most effective to achieve positive DEP trapping.

The local response of PS beads depends on their polarization with respect to the surrounding medium, as represented by Re[*K*(*ω*)].^7^ This dependency changes the response of PS beads at varied frequencies. The number of PS beads through their volume is proportional to the volume of PS beads.^55^ The conductivity of PS beads/liquid interface is usually different from that of bulk liquid or bulk particle, which is usually higher. The conductivity of PS beads is the sum of these volumes and surface contributions. The conductivity of PS beads therefore depends on the surface volume ratio of particles, and usually increasing with the increase of surface volume ratio. The small PS beads (1 μm) of a given material are usually more conductive than large PS beads (10 μm).

The aggregation of PS beads influences the overall behavior of the system, resulting in “less than ideal” separating efficiencies. Nevertheless, in the vicinity of microelectrode tips, this issue was alleviated since the 10 μm PS beads were either repelled toward the outer region of the electric field, or retained above the microelectrodes at low levitation heights, or trapped between the tips. The dimensions of used PS beads are similar to those of prokaryotic (∼1 μm)^56^ and eukaryotic cells (∼10 μm).^4^ The offered system has therefore a great potential for biological applications.

#### 4.3.2. Separating organic and inorganic beads

PS beads, ZnO and silica beads were mixed in Sam-B, and then a drop of mixture suspension was placed on a DEP chip to sort out different particles at various frequencies as illustrated in Fig. 7B. With the applied frequency of 2 kHz, most of the silica particles were dragged to the outer region of micro-electrodes, while PS beads concentrated at the center of micro-electrodes. Moreover, some of the ZnO beads were attracted to the edge and center of micro-electrodes.

Silica beads are expected to have nDEP over 1 kHz, and moved away from micro-electrodes when frequency increased, as illustrated in Fig. 6A. At *f* = 10 kHz, zinc oxide beads were all rotated near the edge of micro-electrodes, while PS beads were still at the center of micro-electrodes, as shown in Fig. 7B(b). When the frequency was increased to 70 kHz, PS beads all moved away from micro-electrodes by nDEP; ZnO beads only stay on the edge of micro-electrodes as shown in Fig. 7B(c). PS beads and non-bioparticles were separated successfully at frequency of 10 kHz (see Fig. 7B.avi in ESI†). Zhao and Li^27^ previously utilized a nano-orifice based microfluidic device to separate PS beads and sliver-coated hollow glass beads by DC DEP (>120 V). Here, organic and inorganic beads (Sam-B) were therefore separated successfully, especially offering certain advantages of being mild process conditions.

The measured crossover frequency of the three types of particles were investigated in Honegger's experiment, but there is no suitable microelectrode chip to separate the three particles at the same time.^38^ According to different crossover frequency, three different types of particles can be separated at the same time in our work.

### 4.4. DEP manipulation of *S. aureus*

#### 4.4.1. Separating *S. aureus* from non-bioparticles

**Fig. 8:**
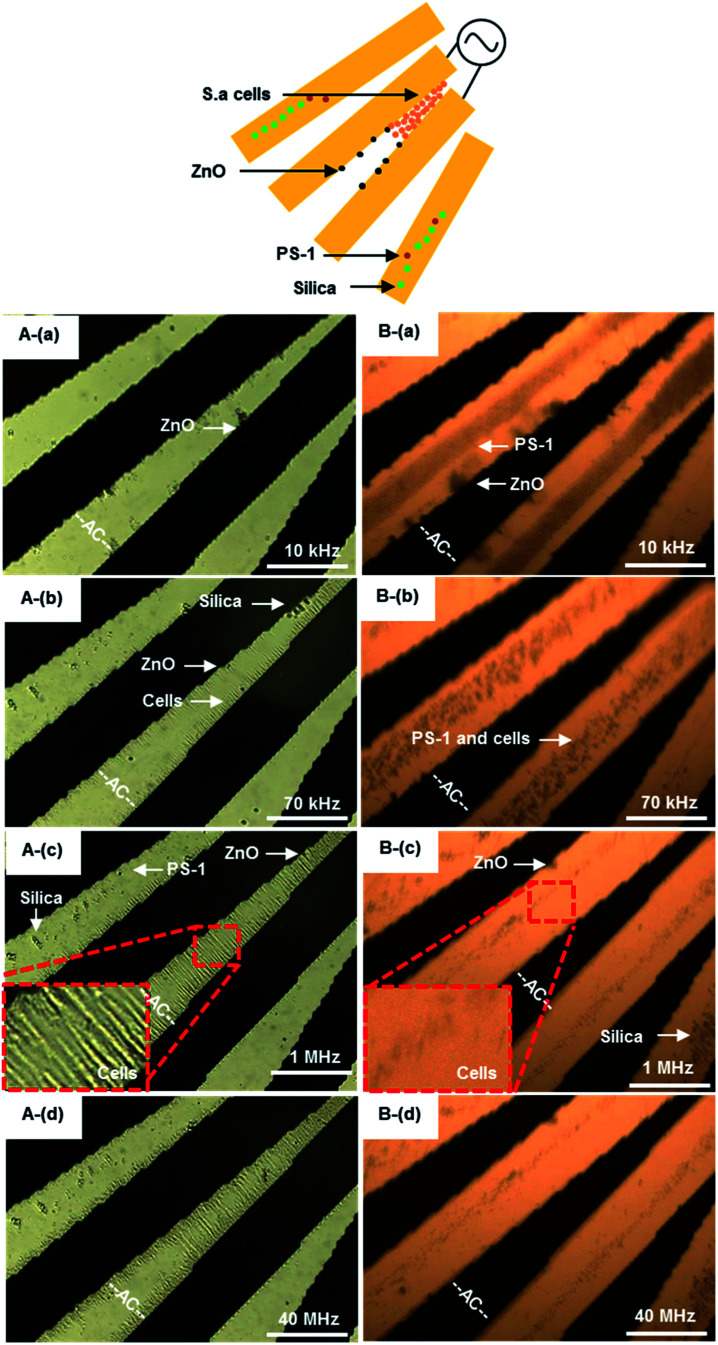
The frequency-sequenced separation of PS beads and *S. aureus* by DEP utilizing transmission (A) and reflection (B) of optical microscopy. The samples with concentrations of 4.6 × 10^7^ particles per mL. The AC signal frequency range 10 kHz to 40 MHz with amplitude of 8*V*_pp_. The effective frequency to separate *S. aureus* cells from non-bioparticles is from 70 kHz to 1 MHz for pDEP. The white scale bar indicates 50 μm. Multimedia view: Fig. 8.avi.† A(a) and B(a): 10 kHz, ZnO beads rotating around the electrode edge of, PS beads concentrated at the electrode center, while silica beads moved away from electrodes. A(b) and B(b): 70 kHz, ZnO beads still rotating around the electrode edge, most of PS beads and all silica beads moved away from electrodes. In contrast, *S. aureus* cells were to attract to the electrode gap. A(c) and B(c): 1 MHz, ZnO beads still rotating around the electrode edge, all of PS and silica beads moved away from electrodes. In contrast, more and more *S. aureus* cells concentrated at the electrode gap. A(d) and B(d): 40 MHz, all of non-bioparticles moved away from electrodes, while some *S. aureus* cells moved away from the gap.

As discussed organic and inorganic beads above, separation or sorting of biological cells could be further achieved by utilizing the localized AC-DEP. Because of the potential of AC-DEP as a front-end method for bacterial analysis in water, it was important to determine the behavior of *S. aureus* in the presence of inert, non-bioparticles of size similar to the bacteria that could be in a sample background. The dielectric responses of the cells are, however, much more complicated because of the diverse dielectric properties of intracellular substances. As indicated in Fig. 1, particles made of non-conducting PS are only nDEP in the frequency range 100 kHz to 2 MHz. An investigation was conducted to characterize 1 μm beads and same-sized *S. aureus* simultaneously using transmission and reflection mode of optical microscopy as shown in Fig. 8.

Beads and bioparticles (1 μm sized live *S. aureus*) were suspended in DI water and separated by varied frequencies. PS beads were trapped at the electrode center if frequency was 10 kHz as shown in Fig. 8A(a) and B(a), while silica beads moved away from micro-electrodes. *S. aureus* cells and ZnO beads were to be concentrated around the edge of micro-electrodes, where the electric field is strongest. *S. aureus* cells started to be concentrated in the gap of micro-electrodes, as shown in Fig. 8A(b). While PS beads were from the center to the edge of micro-electrodes at 70 kHz, as shown Fig. 8B(b). ZnO beads were still at the edge of micro-electrodes. The motion was presented in Fig. 8.avi of ESI.† One of the main reasons could be due to the gravity of ZnO beads being much greater than that of cells, as shown in Table 2. Another possible reason could be due to polarization of cells forming chains with adjacent cells. Up to 1 MHz, *S. aureus* was aligned in the gap (Fig. 8A(c)) and less dense on the micro-electrodes (Fig. 8B(c)), while non-bioparticles moved away from micro-electrodes. More and more *S. aureus* cells were aggregated at the gap. At 1 MHz, *S. aureus* was dominated by pDEP, polarized and distributed at the electrode edge where the electric field is strongest. As a result, *S. aureus* reaches the highest rate of aggregation at the edge of the electrode. As the frequency continues to increase, there is not much increase in *S. aureus* at the edge of the electrode. Finally, *S. aureus* was released at 40 MHz from the gap to the center of micro-electrodes, as indicated in Fig. 8A(d) and B(d).

The frequency of separation *S. aureus* from non-biological beads could be estimated from 70 kHz to 1 MHz. This phenomenon was in good agreement with the results by Shangguan *et al.*,^57^ for which *S. aureus* was enriched between the electrodes when the frequency was from 10 kHz to 10 MHz by pDEP. Here, the images of transmission and reflection provide valuable evidence regarding how *S. aureus* and non-biological beads locate themselves. Trapping usually comes from pDEP, but there is also a contribution from aggregated *S. aureus* cells on top of the micro-electrodes at frequencies if nDEP dominated, as especially shown in Fig. 8B(b). When the frequency above 70 kHz, non-bioparticles controlled by nDEP and moved away from micro-electrodes (see Fig. 8.avi in ESI†).

In summary, this tunable experimental system achieves differential trapping of cells and particles demonstrating the potential of AC-DEP for cell/particle discrimination and concentration. The controllable performance of the systems enables it to sort and retain specific particles from a mixed population.

#### 4.4.2. Distinguishing live and dead *S. aureus* cells

**Fig. 9:**
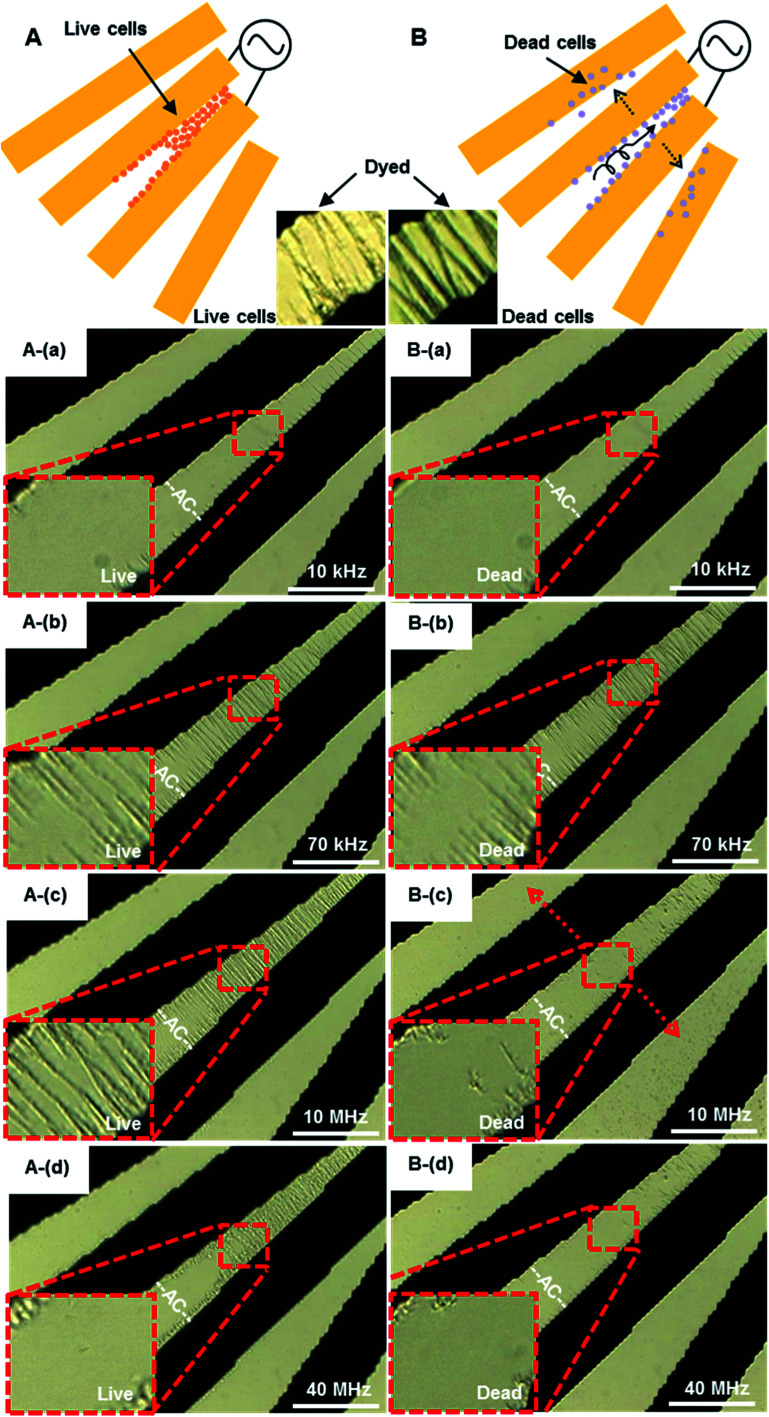
The accumulation and separation of live (A) and dead (B) 4.4 × 10^7^ cells per mL *S. aureus* by pDEP. An AC signal of amplitude 8*V*_pp_ was applied to micro-electrodes over the frequency range from 10 kHz to 40 MHz. (A): (a and b) 10–70 kHz, live cells concentrated at the electrode gap, (c) 10 MHz, some live cells were to move away from the electrode, (d) 40 MHz, most of live cells at gap of electrode. Multimedia view: Fig. 9A.avi† (B): (a and b) 10–70 kHz, dead cells were concentrated at the electrode gap, (c) 10 MHz, most of the dead cells moved away from the gap, (d) 40 MHz, only a few dead cells at the electrode edge. The white scale bar indicates 50 μm. Multimedia view: Fig. 9B.avi.†

Live (A) and dead (B) *S. aureus* cells were respectively introduced into the DEP system. The differential trapping phenomena were observed, as shown in Fig. 9. The DEP collection observations were made at four different electric field frequencies ranging from 10 kHz to 40 MHz. Below 10 MHz, both viable and nonviable bacteria were trapped around the electrode edge due to pDEP as shown in Fig. 9A(a) and B(a), and further aligned in the gap as shown in Fig. 9A(b) and B(b). Both motions were respectively presented in Fig. 9A.avi and B.avi of ESI.† On the other hand, only live bacteria were trapped and aligned by pDEP in the gap as shown in Fig. 9A(c), and dead cells were not effectively collected above 10 MHz as shown in Fig. 9B(c). Dead cells were revolved and moved to both sides of electrodes (outside of the electric field) by nDEP. The pDEP force exerted on dead *S. aureus* cells is negligibly small when the frequency above 10 MHz. The different DEP behaviors of dead and live *S. aureus* cells are due to the conductivities of their cell membranes, as shown in Table 1.

Increasing the frequency to 40 MHz, most of the live cells still stayed in the gap, while dead cells were not concentrated in the gap any more. The DEP force tends to decrease slightly with increasing frequency of the applied field. The midplane of the gaps is the only region over the electrode plane where the DEP force is substantially affected by frequency. The DEP responses of live and dead *S. aureus* cells show a striking contrast at the frequency range from 10 MHz to 40 MHz. These results agree qualitatively with the values of the CM factors shown in Table 1. At low frequencies, the applied electric field is primarily dropped across the outer cellular membrane, and the cells behave as poorly conductive spheres.^55^ At higher frequencies, the applied field is able to penetrate into the cells, and the cells behave more as conductive spheres having the conductivity of the cells' interior. Lapizco-Encinas *et al.*^55^ concentrated live and dead *E. coli* by DC-DEP, and dead cells have significantly lower DEP mobility than live cells. When a cell died, its membrane becomes permeable and the conductivity could increase by a factor of 10^4^. The DEP separation of live and dead cells should be therefore possible, due to the differences of their membrane conductivities. The difference of electrophoresis mobility of dead and living cells in our work is obvious, which is easier to observe than that of other studies.^55^

## 5. Conclusions

**Fig. 10:**
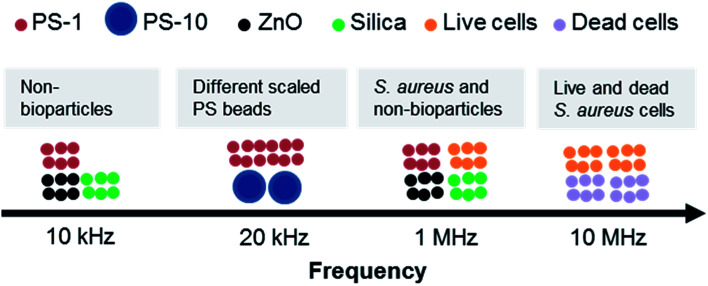
The optimal frequency range of DEP separation.

A novel platform for separating particles according to their dielectric response to AC fields at specific frequencies was presented. The performance of DEP-on-a-chip was simulated using finite element methods by COMSOL. The DEP behaviors of PS beads with varied size, ZnO, silica beads and *S. aureus* cells at different frequencies were characterised, especially optimal frequency for particle separation. The various PS beads were experimentally separated from other non-bioparticles (*e.g.*, ZnO and silica beads) at frequencies of 20 kHz and 10 kHz, respectively, as illustrated in Fig. 10.

When *S. aureus* cells implemented, the optimal frequency range of manipulation was from 70 kHz to 10 MHz by pDEP. In addition, the response of live and dead *S. aureus* cells showed a striking contrast at the frequency range of 10 MHz to 40 MHz. The separation by DEP at moderate frequencies is appropriate not only for separating cells from low dielectric particles, but also for separating cell mixtures. DEP-on-a-chip is expected to be a useful tool for sensing various environmental samples, including cells, proteins, and organic or soil particles, especially integration with highly sensitive quantification of surface acoustic wave.^58^

This electric field configuration is distinguished from the typical coplanar field arrangement by allowing the use of much lower frequencies and applied voltages to pattern and separate particles. The use of mildly applied voltages is favorable for manipulating biological cells. The proposed configuration produces complex non-uniform electric fields, which improve the separation efficiency of multi-particles. In addition, non-bioparticles and cells are positioned in a more precise and controllable manner to form a “strings of pearls”, spanning the gaps between adjoining micro-electrodes. In particular, low frequencies (<100 kHz) offer an advantage of considerably more rapid particle positioning and separation, compared to the results achieved at high frequencies (>100 kHz). The highly electric fields can generate fluid motion, resulting in a viscous drag force on particles.

## Conflicts of interest

There are no conflicts to declare.

## Supplementary Material

RA-010-C9RA05886A-s001

RA-010-C9RA05886A-s002

RA-010-C9RA05886A-s003

RA-010-C9RA05886A-s004

RA-010-C9RA05886A-s005
